# Biofilm formation in *Acinetobacter baumannii* was inhibited by PAβN while it had no association with antibiotic resistance

**DOI:** 10.1002/mbo3.1063

**Published:** 2020-07-22

**Authors:** Lihua Chen, Haixia Li, Haichu Wen, Binyu Zhao, Yujia Niu, Qianqian Mo, Yong Wu

**Affiliations:** ^1^ Department of Laboratory Medicine Third Xiangya Hospital, Central South University Changsha China; ^2^ Department of Medical Laboratory Changsha Medical University Changsha China; ^3^ Department of Laboratory Medicine Xiangya Medical School of Central South University Changsha China

**Keywords:** *Acinetobacter baumannii*, antibiotic resistance, biofilm, efflux pump, PAβN

## Abstract

This study was conducted to investigate the relationship between *Acinetobacter baumannii* biofilm formation and antibiotic resistance. Furthermore, the effects of PAβN, a potential efflux pump inhibitor, on *A. baumannii* biofilm formation and dispersion were tested, and the gene expression levels of efflux pumps were determined to study the mechanisms. A total of 92 *A. baumannii* isolates from infected patients were collected and identified by multiplex PCR. The antimicrobial susceptibility of *A. baumannii* clinical isolates was tested by VITEK 2 COMPACT^®^. Genotypes were determined by ERIC‐2 PCR. Biofilm formation and dispersion were detected by crystal violet staining. The presence and mRNA expression of efflux pump genes were analyzed by conventional PCR and real‐time PCR, respectively. More than 50% of the *A. baumannii* strains formed biofilm and were divided into different groups according to their biofilm‐forming ability. Antibiotic resistance rates among most groups did not significantly differ. There were 7 clonal groups in 92 strains of *A. baumannii* and no dominant clones among the different biofilm‐forming groups. PAβN inhibited *A. baumannii* biofilm formation and enhanced its dispersion, whereas *adeB*, *adeJ,* and *adeG* and the mRNA expression of *adeB*, *abeM*, and *amvA* showed no differences in the different biofilm‐forming groups. In conclusion, there was no clear relationship between biofilm formation and antibiotic resistance in *A. baumannii*. The effects of PAβN on *A. baumannii* biofilm formation and dispersion were independent of the efflux pumps.

## Graphical abstract

We showed that *Acinetobacter baumannii* had a strong biofilm formation ability. This ability was not clearly associated with antibiotic resistance and was inhibited by PAβN. The mechanisms of the effects of PAβN on *A. *baumannii biofilm formation and dispersion may be independent of the efflux pumps.

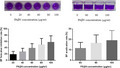

## 1 INTRODUCTION

*Acinetobacter baumannii* is a nonfermentative, oxidase‐negative, gram‐negative coccobacillus. It is also one of the most common bacterial species causing hospital‐acquired infections, including epidemic pneumonia, bloodstream infections, meningitis, and urinary tract infections (Nait et al., 2014). The resistance rate of *A. baumannii* is increasing annually, particularly, the rate of infection with carbapenem‐resistant *A. baumannii* has increased steadily, creating challenges in the clinic (Tacconelli et al., 2018). Therefore, the American Centers for Disease Control and Prevention recognizes *A. baumannii* as a source of global outbreaks and epidemics, specifically because of its increasing resistance to commercially available antibiotics (Handal et al., 2017).

Although infections caused by *A. baumannii* have attracted the attention of researchers, its virulence factors, including biofilm, are not well‐understood (Dahdouh, Hajjar, Suarez, & Daoud, 2016). Compared to its corresponding planktonic bacteria, biofilm‐forming bacteria exhibit a modified phenotype, showing differences in gene transcription and interactions with other bacteria (Steenackers, Parijs, Foster, & Vanderleyden, 2016). Biofilm plays an important role in persistent infections caused by pathogenic microorganisms. It has been reported that 65% of human infections are related to biofilm, which show up to 10‐ to 1,000‐fold higher resistance compared to their corresponding planktonic cells (Cerqueira & Peleg, 2011; Sirijant, Sermswan, & Wongratanacheewin, 2016). Therefore, biofilm‐related infections are more difficult to clear and more prone to relapse (Koo, Allan, Howlin, Stoodley, & Hall‐Stoodley, 2017). Notably, some studies have demonstrated that biofilm formation is increased in response to subinhibitory concentrations of antibiotics, which is typically a direct consequence of low‐dose therapy, indicating that biofilm regulation is involved in the global response to external stresses, such as antibiotics (Kaplan, 2011; Zaborskyte, Andersen, Kragh, & Ciofu, 2017). However, the correlation between the biofilm formation ability of *A. baumannii* and antibiotic resistance remains unclear. Over the past two decades, numerous studies have shown conflicting results (Perez, 2015; Sanchez et al., 2013; Wang et al., 2018), and thus further, studies are needed to resolve these issues.

The mechanisms of antibiotic resistance in *A. baumannii* are very complex, mainly involving the expression of active efflux pumps; changes in antibiotics targets; production of inactive enzymes; and reduction, deficiency, and mutation of outer membrane proteins (Lee et al., 2017). Overexpression of efflux pumps can decrease the accumulation of drugs and may be among the main mechanisms inducing *A. baumannii* multidrug resistance (Lin, Lin, Tu, & Lan, 2017; Yoon et al., 2015). To date, three *Acinetobacter* drug efflux (Ade) resistance–nodulation–cell division (RND) systems, AdeABC (Magnet, Courvalin, & Lambert, 2001), AdeFGH (Coyne, Rosenfeld, Lambert, Courvalin, & Périchon, 2010), and AdeIJK (Damier‐Piolle et al., 2008), have been characterized in *A. baumannii*. In a study of 53 tigecycline‐susceptible *A. baumannii* isolates, the relative expression levels of *adeB* and *adeJ* were significantly increased in 52 clinical tigecycline‐non‐susceptible isolates (Li et al., 2015). Another study showed that highly multidrug‐resistant *A. baumannii* were positive for adeABC and adeIJK (Lin, Ling, & Li, 2009). AbeM, a type of efflux pump, was reported to play an important role in the resistance to imipenem (Hou, Chen, Yan, Wang, & Ying, 2012). AmvA is also efflux pump and belongs to the major facilitator superfamily and mediates the resistance of a clinical multidrug‐resistant *A. baumannii* isolate. The expression of AmvA was found to be higher in clinical isolates that exhibited very high MICs of carbapenems, cephalosporins, aminoglycosides, and fluoroquinolones (Rajamohan, Srinivasan, & Gebreyes, 2010). Most studies of efflux pumps have focused on their roles in increasing antibiotic resistance. The relationship between these pumps and biofilm formation requires further analysis. Phenylalanine‐arginine beta‐naphthylamide (PAβN) is a kind of efflux pump inhibitor. PAβN has been proposed to be an RND competitive substrate (Kourtesi et al., 2013). RND‐type multidrug efflux pumps are a critical contributor to antibiotic resistance in *A. baumannii* (Leus et al., 2018). The effects of the PAβN on biofilm formation and dispersion of *A. baumannii* remain unknown.

In this study, the associations among the biofilm formation in 92 nonduplicated *A. baumannii* strains isolated from infected patients and antibiotic resistance were investigated. Additionally, the effects and possible mechanisms of PAβN on *A. baumannii* biofilm formation and dispersion were examined.

## 2 MATERIALS AND METHODS

### 2.1 Bacterial isolates and data collection

The clinical isolates used in this study were collected from June 2014 to January 2015 as different kinds of specimen sample types, including sputum, urine, abscess secretion, blood, hydrothorax, and ascites, from infected patients who visited the Third Xiangya Hospital of Central South University, P.R. China, a three A‐level large‐scale comprehensive teaching hospital with 2,200 beds. Bacterial strains were primarily identified as *A. baumannii complex* by VITEK 2 COMPACT^®^ automatic system (bioMérieux). Bacterial cultures were preserved in bacterial magnetic bead cryopreservation tubes, maintained at −80°C, and subcultured on blood agar plates overnight to obtain monoclonal colonies at 37°C before each experimental assay. The antibiotic susceptibility of all strains was evaluated using the VITEK 2 COMPACT^®^ automatic system and determined according to Clinical and Laboratory Standards Institute procedures (Clinical & Laboratory Standards Institute, 2014). *Pseudomonas aeruginosa* ATCC 27853 and *Staphylococcus aureus* ATCC 27923 served as quality controls for the susceptibility test.

### 2.2 DNA extraction by boiling and freeze‐thawing method

*Acinetobacter baumannii* genomic DNA was extracted without using chemical reagents and DNA purification. The frozen strain was transferred to a blood agar plate and incubated aerobically at 37°C overnight after which it was transferred to another blood agar plate to obtain the third generation. The third generation was used to extract genomic DNA. Approximately 10 single colonies of bacteria were selected and transferred into a microtube containing 400 μl ddH_2_O. The microtube was incubated in boiling water for 10 min, immediately cooled on ice, and frozen at −20°C for 20 min. The samples were then thawed at room temperature (approximately 25°C) and homogenized by vortex mixing for 10 s. Finally, the microtubes were centrifuged at 13,362 *g* for 15 min at 4°C. The upper aqueous layer containing the DNA was carefully transferred to a sterile microtube. The DNA samples were stored at −80°C until use.

### 2.3 Identification of *A. baumannii* by multiplex polymerase chain reaction

We collected the strains first identified as *A. calcoaceticus– A. baumannii complex* with the VITEK 2 COMPACT^®^ automated microbiology system. One‐tube multiplex polymerase chain reaction (PCR) was performed as described previously to isolate *A. baumannii* (Chen et al., 2007). A pair of primers was used to amplify an internal 208‐base pair (bp) fragment from the internal transcribed spacer (ITS) region of *A. baumannii* and the second pair of primers to amplify a highly conserved 425‐bp region of the recA gene of *Acinetobacter* spp. PCR was performed in a total reaction volume of 25 μl containing 12.5 μl 2 × Taq Master Mix, 8.5 μl ddH_2_O, 1 μl each primer for the ITS (10 μM), and 0.5 μl recA (10 μM), with 1 μl DNA template. The PCR conditions were as follows: 94°C for 3 min, followed by 30 cycles of 94°C for 30 s, 55°C for 30 s, and 72°C 30 s, with a final extension at 72°C for 3 min. Amplicons were analyzed by 1.5% agarose gel electrophoresis in 0.5× Tris/boric acid/EDTA buffer run at 100 V for 35 min.

### 2.4 Quantification of the biofilm‐forming ability of *A. baumannii*

Overnight cultures were used to inoculate in 5 ml of LB broth (Qi et al., 2016; Rodríguez‐Baño et al., 2008), which was grown again overnight. The cultures were centrifuged, and the supernatant was discarded. Normal saline was added to the bacterial precipitate and adjusted to 0.5 McFarland standard. Each of six wells of a sterile 96‐well polystyrene microtiter plate was filled with 50 μl of the culture and 150 μl of fresh LB broth. *A. baumannii* type strain ATCC 19606 was used as a positive control, while uninoculated LB media was used as a negative control. The plates were covered and aerobically incubated at 37°C for 48 hr. Next, the plates were gently washed with 1× phosphate‐buffered saline (pH 7.4) 3 times and stained with 100 μl of 0.1% crystal violet (Sigma‐Aldrich) for 15 min at room temperature. The biofilm was quantified by measuring the corresponding OD_570_ of the supernatant following solubilization with crystal violet in 95% ethanol.

The ability of the isolates to form biofilm was classified into four categories as follows: (1) OD ≤ ODc = negative; (2) ODc < OD ≤ 2ODc = weakly positive; (3) 2ODc < OD ≤4ODc = positive; and (4) 4OD < ODc = strongly positive. ODc indicates the mean OD value of the control wells, whereas OD is the value of the tested isolates (Stepanović, Vuković, Dakić, Savić, & Švabić‐Vlahović, 2000).

### 2.5 Homology analysis of *A. baumannii* by enterobacterial repetitive intergenic consensus sequence type 2‐based PCR

The homology of the isolates was evaluated by enterobacterial repetitive intergenic consensus sequence type 2‐based PCR (ERIC‐2 PCR), which reveals strain‐specific banding patterns obtained by amplifying multiple anonymous regions of the genome with the primer ERIC‐2, as previously described by Versalovic, Koeuth, and Lupski (1991). Template DNA was extracted as described in section 2.2. The PCR system contained 12.5 μl 2× Taq Master Mix, 10.5 μl ddH_2_O, 1 μl ERIC‐2 primer (10 μM), and 2 μl DNA template. The reaction mixtures were amplified as follows: initial denaturation at 94°C for 3 min, followed by 30 cycles of DNA denaturation at 94°C for 1 min, annealing at 54°C for 1 min, primer extension at 72°C for 1 min, and a final extension step at 72°C for 10 min. The amplification products were electrophoresed in 1.8% agarose gels, stained with Gelstain, and photographed under UV light. The results were analyzed by visual inspection. Isolates were considered as a single clonal group when they exhibited at least one high‐intensity band difference according to visual inspection.

### 2.6 Presence of efflux pump genes *adeB*, *adeJ*, and *adeG* analyzed by conventional PCR

Fifteen isolates were chosen from each group to detect the positive rates of the efflux pump genes *adeB*, *adeJ*, and *adeG*. DNA templates were extracted as described in section 2.2. PCR amplification was performed in a 25 μl reaction mixture, containing 12.5 μl 2× Taq Master Mix; 10.5 μl ddH_2_O; 0.5 μl each primer for *adeB*, *adeJ*, and *adeG* (Table 2); and 1 μl DNA template. The primers used for conventional PCR analysis are listed in Table 1. The PCR conditions were as follows: 94°C for 3 min, followed by 30 cycles of 94°C for 30 s, 60°C for 30 s, and 72°C for 30 s, with a final extension at 72°C for 3 min. Amplicons were analyzed by electrophoresis in a 1.5% agarose gel in 0.5× Tris/boric acid/EDTA buffer at 100 V for 35 min.

**TABLE 1:** Nucleotide sequence of the specific primers used in PCR amplification

Primer	Sequence (5′ to 3′)	References
recA‐F	CCTGAATCTTCTGGTAAAAC	Chen et al. (2007)
recA‐R	GTTTCTGGGCTGCCAAACATTAC	
ITS‐F	CATTATCACGGTAATTAGTG	Chen et al. (2007)
ITS‐R	AGAGCACTGTGCACTTAAG	
ERIC‐2	AAGTAAGTGACTGGGGTGAGCG	Versalovic et al. (1991)
16S rRNA‐F	GACGTACTCGCAGAATAAGC	Lin et al. (2009)
16S rRNA‐R	TTAGTCTTGCGACCGTACTC	
adeB^1^‐F	TTAACGATAGCGTTGTAACC	Lin et al. (2009)
adeB^1^‐R	TGAGCAGACAATGGAATAGT	
adeG‐F	TTCATCTAGCCAAGCAGAAG	Coyne et al. (2010)
adeG‐R	ATGTGGGCTAGCTAACGGC	
adeJ‐F	ATTGCACCACCAACCGTAAC	Lin et al. (2009)
adeJ‐R	TAGCTGGATCAAGCCAGATA	
rpoB‐F	ATGCCGCCTGAAAAAGTAAC	Rumbo et al. (2013)
rpoB‐R	TCCGCACGTAAAGTAGGAAC	
adeB^2^‐F	CGAGTGGCACAACTAGCATC	Rumbo et al. (2013)
adeB^2^‐R	CCTTGTCTTGGCTGCACTCT	
abeM‐F	GTAGGTGTAGGCTTATGGA	Rumbo et al. (2013)
abeM‐R	AAACTGGCTTTAGGTTGTA	
amvA‐F	GCAGAGAAATTTTGCACTTGG	Rumbo et al. (2013)
amvA‐R	CGACTAATGGACCAAAAGCTG	

Note

adeB^1^ was the primer for conventional PCR, and adeB^2^ was the primer for real‐time PCR.

### 2.7 RNA extraction and cDNA synthesis

According to the results of homology analysis, 1–2 isolates were chosen from one clonal group in each biofilm formation ability group for RNA extraction and cDNA synthesis. A single colony‐forming unit of *A. baumannii* was selected from the overnight cultures and inoculated into 5 ml LB and cultured at 37°C and 180 rpm overnight. Next, 1 ml of this culture was centrifuged. RNA extraction was performed using the E.Z.N.A Total RNA Kit (Omega Biotek) following the manufacturer's instructions. Total RNA (1 μg) was used to synthesize cDNA using the EasyScript First‐Strand cDNA Synthesis SuperMix Kit (TransGen Biotech) following the manufacturer's instructions. The synthesized cDNA was stored at −20°C.

### 2.8 Quantitative reverse transcriptase–PCR

Three different efflux pump‐encoding genes, *adeB*, *abeM*, and *amvA,* were analyzed by quantitative reverse transcriptase–PCR (qRT‐PCR). The primers used for qRT‐PCR analysis are listed in Table 1. Gene expression was determined by qPCR using the TransStart SYBR Green qPCR SuperMix UDG Kit (TransGen Biotech). A no‐template control was included in the analysis. *RpoB* was used as the reference gene. Relative expression was determined using the 2^−ΔΔCt^ method with the Eppendorf Real‐Time System. Reaction samples were prepared according to the manufacturer's instructions with a 10‐fold dilution of cDNA. All reactions were carried out in triplicate with at least two biological replicates. Target gene expression was measured as the relative expression compared to that of *rpoB*.

### 2.9 Assessment of the effects of PAβN on *A. baumannii* biofilm formation and dispersion

To determine the relationship between biofilm formation ability and efflux pump expression, the effects of PAβN, a potential efflux pump inhibitor, on *A. baumannii* biofilm formation and dispersion were examined. The method was adapted from a previously described study (Chen et al., 2016). PAβN at final concentrations of 0, 20, 40, 60, 80, or 100 μg/ml was cocultured with 10 strains of *A. baumannii* with biofilm formation ability in a 96‐well cell culture microtiter plate for 24 hr. The cells were stained with crystal violet, and the OD_570_ was measured. To evaluate the dispersion effect, the strains were cultured to primarily form biofilm in a 96‐well cell culture microtiter plate for 24 hr. The plate was washed with phosphate‐buffered saline, and 200 μl of media containing 0, 60, 80, or 100 μg/ml PAβN in LB was added to each well and incubated for an additional 24 hr. The cells were washed as described above, and the biomass was determined by measuring the OD_570_ with 1% (w/v) crystal violet solubilized in ethanol. Each assay was performed in triplicate.

### 2.10 Statistical analysis

The quantitative data are presented as the means ± standard deviations. Different groups were compared by one‐way analysis of variance followed by Tukey–Kramer multiple comparison test. *p*‐values < .05 were considered as statistically significant. For the antibiotic susceptibility ratios and conventional PCR results, statistical analysis was performed using the chi‐square test. Statistical analysis was conducted using SPSS 21.0 software (SPSS, Inc.).

## 3 RESULTS

### 3.1 *Acinetobacter baumannii* identification by one‐tube multiplex PCR

**FIGURE 1:**
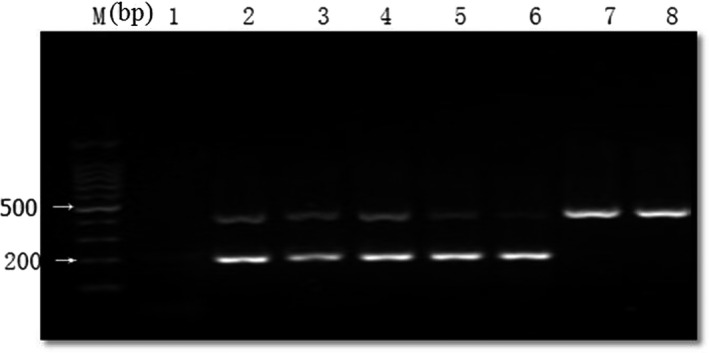
Examples of multiplex PCR products resolved by agarose gel electrophoresis. Note: PCR was performed using the specific *Acinetobacter baumannii* primers for the *ITS* gene (product: 208bp) and the internal control primers specific for the *recA* gene (product: 425bp) of all Acinetobacter spp. Lane M: 100‐bp DNA size ladders; lane 1: *Pseudomonas aeruginosa* ATCC 27853 as negative control; lane 2: the reference strain *A. baumannii* ATCC 19606 as a positive control; lanes 3 ~ 6: the clinical isolates that were identified as *A. baumannii*; lanes 7 ~ 8: the clinical isolates that were identified as other *Acinetobacter* spp.

A total of 121 isolates of *A. calcoaceticus*‐*A. baumannii* (Ac‐Ab) complex, which were identified phenotypically by the VITEK 2 COMPACT^®^ automatic system, were examined by one‐tube multiplex PCR. The reference strain (*A. baumannii* ATCC 19606) and clinical isolates all yielded a 425‐bp internal control amplicon corresponding to *recA*, whereas only *A. baumannii* isolates yielded the 208‐bp fragment of the ITS region (Figure 1). The bacteria belonging to other genera produced no amplicons, including *P. aeruginosa*. We identified 92 isolates belonging to *A. baumannii* among the 121 isolates.

### 3.2 Biofilm formation ability of identified *A. baumannii* and different sample types

Using 96‐well cell culture microtiter plates with crystal violet staining, 50 isolates of the 92 clinical strains of *A. baumannii* showed positive results for biofilm formation. The semiquantitative results of the biofilm formation ability are shown in Table 2.

**TABLE 2:** The biofilm formation ability of the identified *Acinetobacter baumannii*

Biofilm formation ability	Isolates number	Percentage	OD_570_ (mean ± *SD*)
Negative	42	45.65	0.11 ± 0.06
Weak positive	15	16.30	0.26 ± 0.07
Positive	17	18.48	0.43 ± 0.07
Strong positive	18	19.57	0.85 ± 0.28

*Acinetobacter baumannii* originated from different types of samples, all of which exhibited biofilm formation ability. For example, in 74 isolates of *A. baumannii* from sputum samples, 40 were biofilm‐positive (54.04%); 5 of 8 isolates from secretion samples showed biofilm formation ability (62.50%); in 10 isolates from other sample types (blood, hydrothorax, ascites, etc.), 5 isolates could form a biofilm (50.00%).

### 3.3 Antibiotic susceptibility analysis among different biofilm‐forming groups

*Acinetobacter baumannii* in each group showed high resistance rates (>70%) to the most widely used antibiotics in the clinic. Most of the studied antibiotics showed no significant differences in resistance rates among the different groups. Only tobramycin and gentamicin showed significant differences among the different groups, with the lowest resistance rate observed in the biofilm‐forming positive group (Table 3).

**TABLE 3:** The antibiotic resistance ratio in different biofilm formation ability groups

Antibiotics	Negative (*n* = 42)	Weak positive (*n* = 15)	Positive (*n* = 17)	Strong positive (*n* = 18)	Total (*n* = 92)	*p*‐value
Aztreonam	88.10%	80.00%	70.59%	88.89%	83.70%	.225
Ciprofloxacin	88.10%	80.00%	70.59%	83.33%	82.61%	.095
Ceftriaxone	88.10%	73.33%	76.47%	83.33%	82.61%	.42
Cefepime	88.10%	73.33%	70.59%	83.33%	81.52%	.215
Gentamicin	88.10%	86.67%	52.94%*	83.33%	80.43%	*.02*
Imipenem	88.10%	80.00%	64.71%	77.78%	80.43%	.137
Levofloxacin	78.57%	60.00%	64.71%	66.67%	70.65%	.408
SXT	76.19%	73.33%	76.47%	77.78%	76.87%	.964
Tobramycin	88.64%	73.33%	47.06%*	83.33%	77.17%	*.013*
TZP	76.00%	68.75%	41.18%	77.78%	68.48%	.175
Tigecycline	0.00%	13.33%	5.88%	16.67%	6.52%	.108

Abbreviations: SXT, trimethoprim–sulfamethoxazole; TZP, piperacillin/tazobactam.

Italics indicates the value of *p* < 0.05.

* *p* < 0.05 versus negative control group.

### 3.4 Homology analysis of *A. baumannii* by ERIC‐2 PCR

**FIGURE 2:**
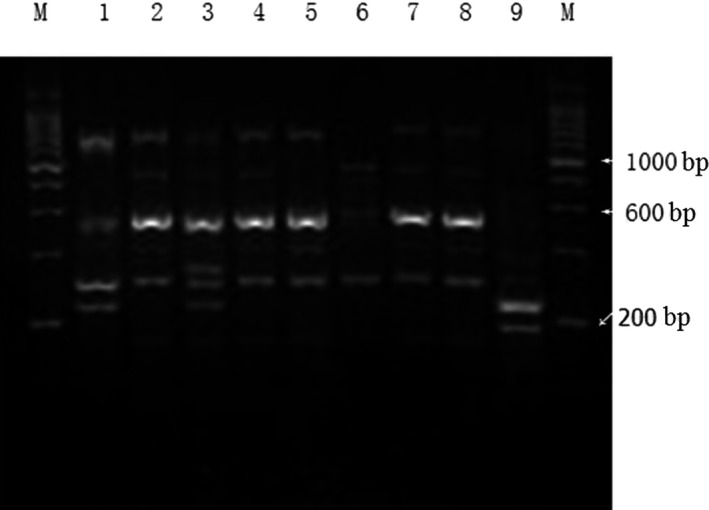
The genotyping result of the studied *Acinetobacter baumannii* by ERIC‐2 PCR. Note: M was 200‐bp DNA ladder; 1 standard for *A. baumannii* ATCC 19606; 2 belonged to genotype A, 3 was genotype B; 4, 5, 7, and 8 were the same genotype C; 6 was genotype D; and 9 belonged to genotype E

The clonal relationships of 92 *A. baumannii* isolates were obtained by ERIC‐2 PCR and found to have 7 different genotypes. Figure 2 shows the results of ERIC‐2 PCR analysis for genotypes A‐E. Among the studied isolates, 42 isolates in the biofilm‐negative group showed 5 genotypes, 15 isolates in the weak positive group showed 5 genotypes, and 17 isolates in the positive group showed 6 genotypes; 18 isolates in the strong positive group showed 6 genotypes, whereas the fingerprint of AB ATCC 19606 differed from that of the clinical isolates (Figure 2). The biofilm‐positive isolates were distributed among these 7 genotypes and exhibited no obvious central trends and regularity.

### 3.5 Presence and expressions of efflux genes in different biofilm‐forming groups

**FIGURE 3:**
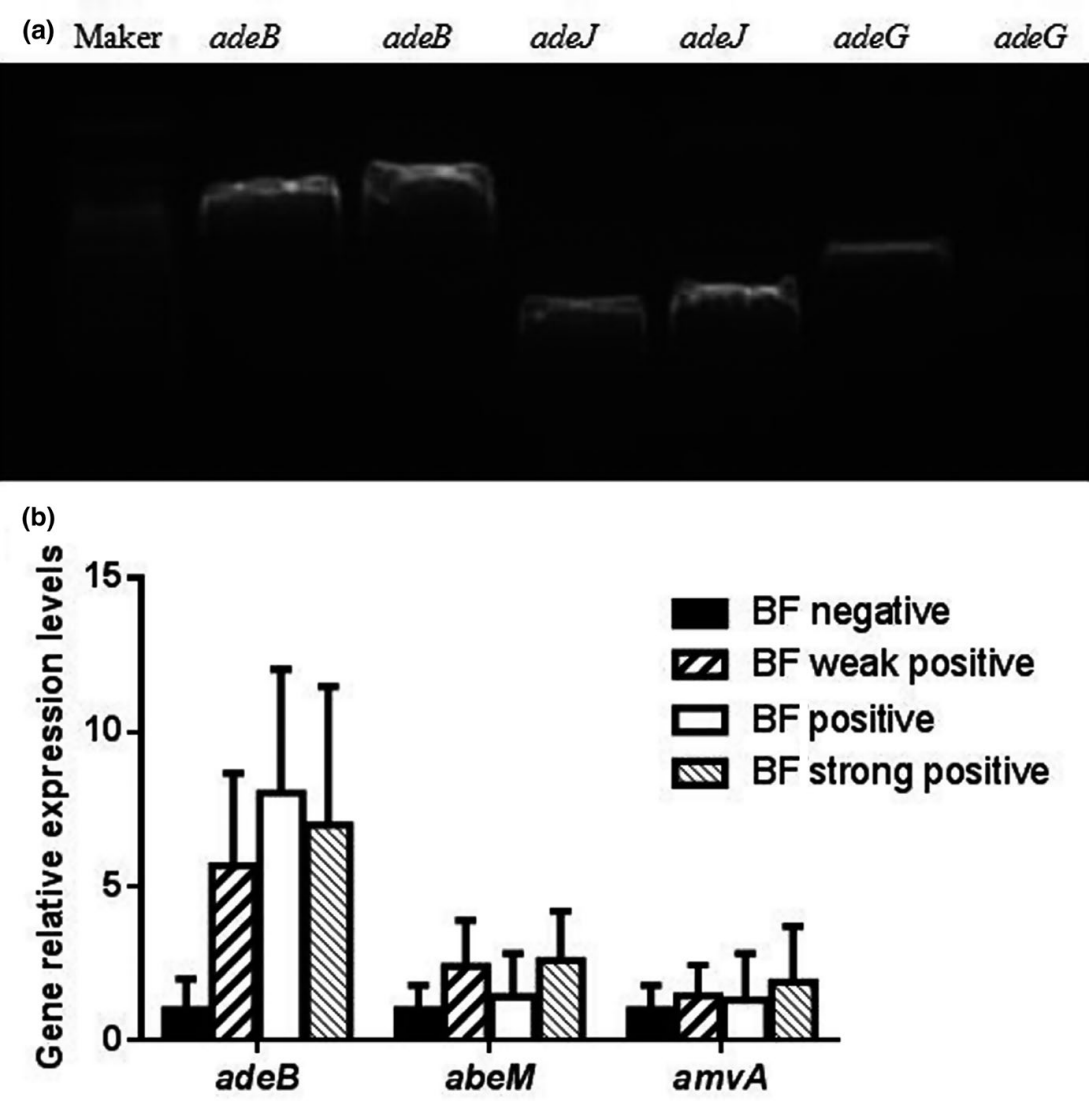
Example of the conventional PCR result for *adeB*, *adeJ,* and *adeG* of two isolates (a) and the gene relative expression levels of *adeB*, *abeM,* and *amvA* in each group (b)

The distribution of efflux genes *adeB*, *abeJ*, and *adeG* is shown in Table 4. These efflux genes were expressed in most clinic isolates. However, there were no significant differences among the different biofilm‐forming groups. To verify these results, we chose *adeB* and the efflux genes *abeM* and *amvA* for quantification by real‐time PCR. According to the genotyping results of ERIC‐2 PCR, 1–2 strains were selected from each genotype in each group to be tested. The results agreed with those of conventional PCR. The expression levels of *adeB*, *abeM*, and *amvA* did not differ among the four studied groups (Figure 3).

**TABLE 4:** The distribution of efflux genes in different biofilm formation ability groups (*n* = 15 for each group)

	Negative group (%)	Weak positive (%)	Positive (%)	Strong positive (%)	*p*‐value
*adeB*	80	93.33	82.67	86.67	.273
*adeJ*	86.67	100	100	93.33	.285
*adeG*	86.67	93.33	93.33	86.67	.868

### 3.6 Effects of PAβN on *A. baumannii* biofilm formation and dispersion

**FIGURE 4:**
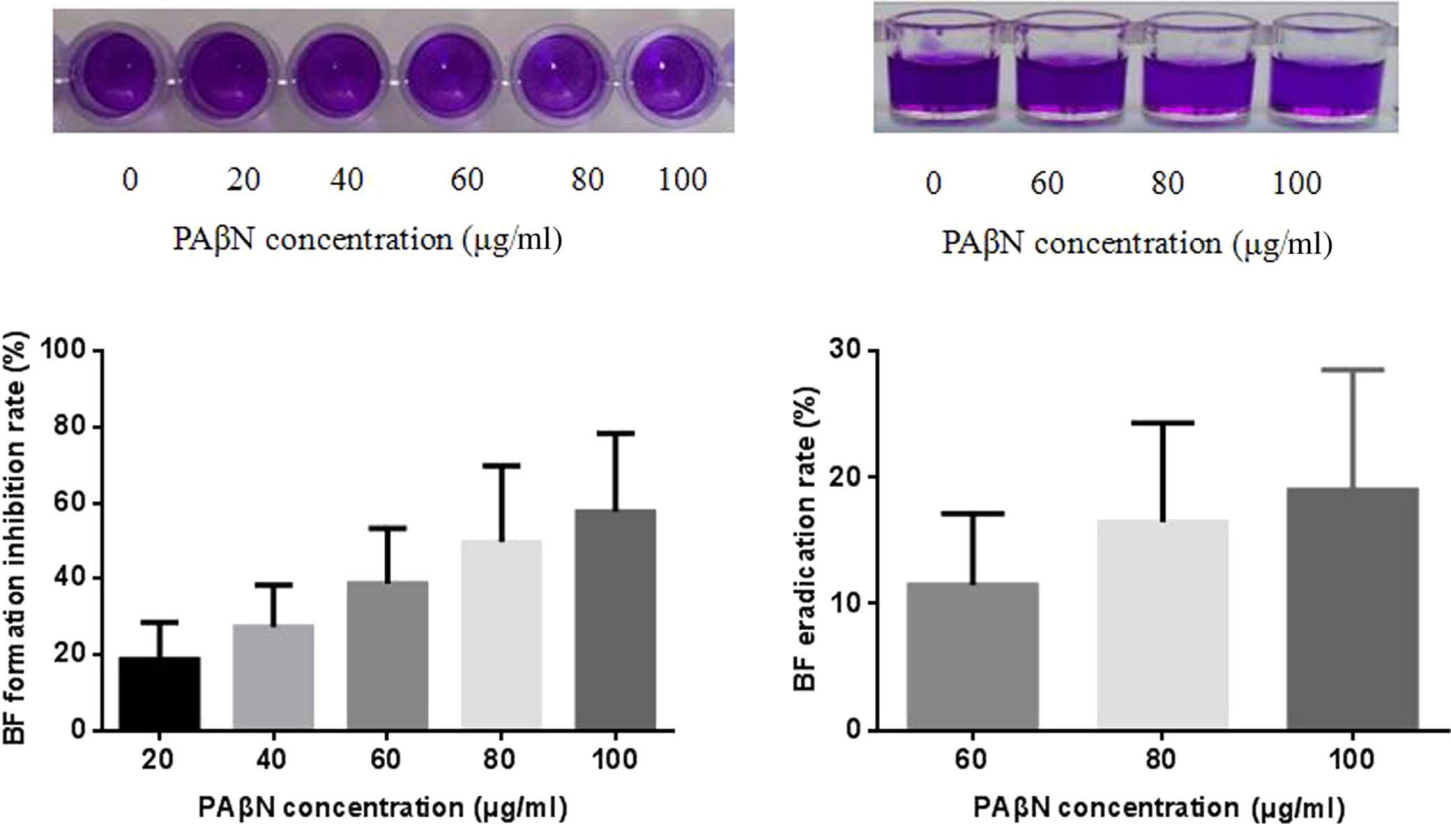
The effect of efflux pump inhibitor PAβN on *Acinetobacter baumannii* biofilm formation (left) and eradication (right). The upper two figures were the samples of PAβN on clinic isolates

The effects of the efflux pump inhibitor PAβN on 9 strains showing strong positive biofilm formation abilities were evaluated. As shown in Figure 4, PAβN significantly inhibited the biofilm formation of the studied isolates in a dose‐dependent manner. PAβN at 100 μg/ml inhibited biofilm formation by 57.71%. PAβN also showed weak eradication effect of the formed biofilm; 100 μg/ml PAβN eradicated 19% of the formed biofilm.

## 4 DISCUSSION

*Acinetobacter baumannii* is widely found on environmental surfaces and is likely important in disease transmission within healthcare settings (Chen et al., 2017). In recent years, *A. baumannii* has attracted attention because of its increased rate of causing serious infections and outbreaks in clinics. In the genus *Acinetobacter*, *A. baumannii*, *Acinetobacter calcoaceticus*, *Acinetobacter* genomic species 3, and *Acinetobacter* genomic species 13 show high levels of genotypic and phenotypic similarity, making them difficult to be distinguished in most routine clinical diagnostic laboratories. Therefore, they are grouped as the *A. calcoaceticus–A. baumannii* (Ac‐Ab) complex (Gerner‐Smidt, Tjernberg, & Ursing, 1991). However, in the Ac‐Ab complex, different genomic species show different antibiotic susceptibilities and epidemic potential. It is very important to rapidly and accurately distinguish *A. baumannii* from other species in the complex. In the present study, using the multi‐PCR method, *A. baumannii* was identified quickly and accurately. Our results showed that in the clinic, the isolates of the Ac‐Ab complex mostly contained *A. baumannii* (76.03%). Additionally, the antibiotic resistance rate was significantly higher than that of other species in the complex (data not shown).

In this study, among the detected 92 strains of *A. baumannii*, 50 strains formed biofilm. In these 50 strains, the biofilm formation abilities were weakly positive, positive, and strongly positive, with approximately 1/3 of the strains in each group. It must be pointed out that although the negative controls were uninoculated LB, its readings were a little high in this study. We hypothesize this was due to unavoidable dye residue rather than contamination, as the procedures for negative control wells were the same as samples, including incubation, washing, staining with crystal violet, and solubilization with ethanol. After all these procedures, the negative controls could not be colorless, which resulted in little high OD readings. The biofilm‐forming abilities of *A. baumannii* strains were determined by the OD value differences between the sample wells and the negative control wells. Interestingly, we observed a relationship between the *A. baumannii* biofilm formation ability and its antibiotic resistance. For most antibiotics, the antibiotic resistance rates were lowest in the positive biofilm‐forming group. However, overall, there was no clear association between biofilm‐forming ability and antibiotic resistance. The association of biofilm formation ability and antibiotic resistance is controversial. Perez reported that in 116 strains of *A. baumannii*, the biofilm formation abilities of meropenem‐resistant strains were significantly lower than those of meropenem susceptibility strains (*p* < .001) (Perez, 2015). Cusumano also reported that multidrug‐resistant *Klebsiella pneumoniae* isolates were more commonly weak than strong in biofilm formation ability (Cusumano et al., 2019). In contrast, another study showed that *A. baumannii* biofilm formation‐positive strains more easily developed a multidrug‐resistant phenotype. Compared to strains with weak or non‐biofilm formation abilities, *A. baumannii* clinic isolates with strong biofilm formation abilities more easily developed resistance to aminoglycosides, carbapenems, tetracyclines, and sulfonamides (Sanchez et al., 2013). In previous studies, the biofilm formation abilities of the chosen strains were not subdivided, which may have led to inconsistent results.

Because biofilm formation and the efflux pump system are both related to *A. baumannii* resistance and survival in the hospital environment, we examined the relationship between biofilm and the efflux pump system. Studies of biofilm and the efflux pump system are limited and have shown controversial results. One study showed that as the *A. baumannii* biofilm matured, *adeB* and *adeG* expression was increased (He et al., 2015). In contrast, another study reported that in a total sequenced *A. baumannii*‐sensitive strain BM4587, overexpressions of AdeABC and AdeIJK significantly reduced biofilm formation (Yoon et al., 2015). Our results revealed no significant differences in these efflux pump genes among strains with different biofilm formation abilities. In general, biofilm is considered as a self‐protective mechanism, as the biofilm forms a barrier to resist various stresses. According to our results for biofilm formation and antibiotic susceptibility, these differences may be because the resistance rate and gene expression were tested in planktonic cultures in vitro but not in biofilm cultures. In the clinic, bacteria may show susceptibility to drugs in vitro but the curative effects are poor in vivo, which may be related to the bacteria's biofilm formation ability in vivo.

Although no efflux pump genes showed a direct relationship with the biofilm formation ability, we investigated the effect of PAβN, a universal efflux inhibitor, on biofilm formation and dispersion. As the ability of *A. baumannii* isolates to acquire drug resistance by the efflux pump mechanism is a concern, most studies of PAβN have focused on antimicrobic susceptibility changes. PAβN was found to inhibit the ability of the AdeFGH pump to efflux trimethoprim, chloramphenicol, and clindamycin in *A. baumannii* strains (Cortez‐Cordova & Kumar, 2011). The minimum biofilm eradication concentration in *Burkholderia pseudomallei* biofilms with ceftazidime and doxycycline was decreased by twofold to 16‐fold in the presence of PAβN (Sirijant et al., 2016). We found that although efflux pump genes did not differ in the different biofilm formation ability groups, PAβN still effectively inhibited biofilm formation and enhanced biofilm dispersion (Figure 4). This observation agrees with data from a previous study, in which PAβN was paired with the iron chelators 2,2′‐dipyridyl, acetohydroxamic acid, and EDTA, which all inhibited *P. aeruginosa* growth and biofilm formation (Liu, Yang, & Molin, 2010). The effects of PAβN on biofilm in this study may be independent of its effect on efflux pumps. For example, in *P. aeruginosa*, PAβN significantly (*p* < .01) decreased the expression of the QS cascade (lasI, lasR, rhlI, rhlR, pqsA, and pqsR) and QS‐regulated type II secretory genes lasB (elastase) and toxA (exotoxin A). Additionally, PAβN eliminated the expression of pelA (exopolysaccharides) (El‐Shaer, Shaaban, Barwa, & Hassan, 2016). The QS system is the main factor involved in regulating biofilm formation by *P. aeruginosa* (Fazli et al., 2014; Rasamiravaka, Labtani, Duez, & El Jaziri, 2015), and pelA is an important biofilm constituent (Marmont et al., 2017). Whether the effects of PAβN on *A. baumannii* biofilm are related to the QS system requires further analysis.

The limitations of this study were its small sample size and retrospective nature. Thus, prospective studies of larger sample sizes are needed to confirm our conclusions. Another limitation is the methodology for biofilm quantification. Until now, there is still no universally recognized standard for biofilm quantification. One report used the reference strain (ATCC 19606) to categorize weak or strong biofilm formers (Qi et al., 2016). Other reports used OD_570_ = 1 to categorize weak or strong/moderate biofilm formation (Beganovic, Luther, Daffinee, & LaPlante, 2019). Another report used the same standard as us (Amin et al., 2019; Zhang et al., 2016)**.** The different standards make it very difficult to compare the studies.

In summary, we showed that *A. baumannii* had a strong biofilm formation ability. Biofilm formation by *A. baumannii* was not associated with antibiotic resistance and was inhibited by PAβN. The mechanisms of the effects of PAβN on *A. baumannii* biofilm formation and dispersion may be independent of the efflux pumps.

## CONFLICT OF INTEREST

None declared**.**

## AUTHOR CONTRIBUTION

**Lihua Chen:** Conceptualization (equal); Funding acquisition (equal); Resources (equal); Writing‐original draft (lead); Writing‐review & editing (equal). **Haixia Li:** Data curation (equal); Formal analysis (equal); Investigation (equal); Validation (equal). **Haichu Wen:** Data curation (equal); Formal analysis (equal); Investigation (equal); Writing‐review & editing (supporting). **Binyu Zhao:** Investigation (supporting). **Yujia Niu:** Investigation (supporting). **Qianqian Mo:** Investigation (supporting). **Yong Wu:** Conceptualization (equal); Funding acquisition (equal); Project administration (lead); Resources (equal); Supervision (lead); Writing‐original draft (supporting); Writing‐review & editing (equal).

## Data Availability

All data generated or analyzed during this study are included in this published article.
